# Case Report: A case of ultrashort wave therapy for suppurative auricular perichondritis secondary to surgery for chronic otitis media

**DOI:** 10.3389/fsurg.2026.1846595

**Published:** 2026-05-18

**Authors:** Miaowei Wang, Dae-Keun Jeong

**Affiliations:** 1 Department of Rehabilitation Medicine, West China Hospital, Sichuan University, Chengdu, Sichuan, China; 2 Department of Physical Therapy, Sehan University, Mokpo, Republic of Korea

**Keywords:** auricular perichondritis, case report, chronic otitis media, surgery, ultrashort wave therapy

## Abstract

The treatment of suppurative auricular perichondritis secondary to surgery for chronic otitis media is characterized by prolonged duration and complexity. Combining the use of physiotherapy alongside surgery and antimicrobial therapy enhances therapeutic efficacy and shortens recovery time. A multidisciplinary approach warrants wider adoption for this disease.

## Introduction

Suppurative auricular perichondritis is an acute suppurative inflammation of the auricular perichondrium. This condition progresses rapidly and may ultimately result in auricular deformity due to compromised blood supply to the auricular cartilage (1). It is mostly caused by trauma to the auricle, surgical procedures, or the spread of infection from adjacent tissues, with bacterial infection being the most frequent cause (2). It is rarely caused by otitis media surgery. The current treatment primarily involves surgical drainage combined with antimicrobial therapy, while physiotherapy is uncommon. Here, we report a case of ultrashort wave therapy as an adjunct treatment for suppurative auricular perichondritis secondary to chronic otitis media surgery.

## Case report

**Figure 1:**
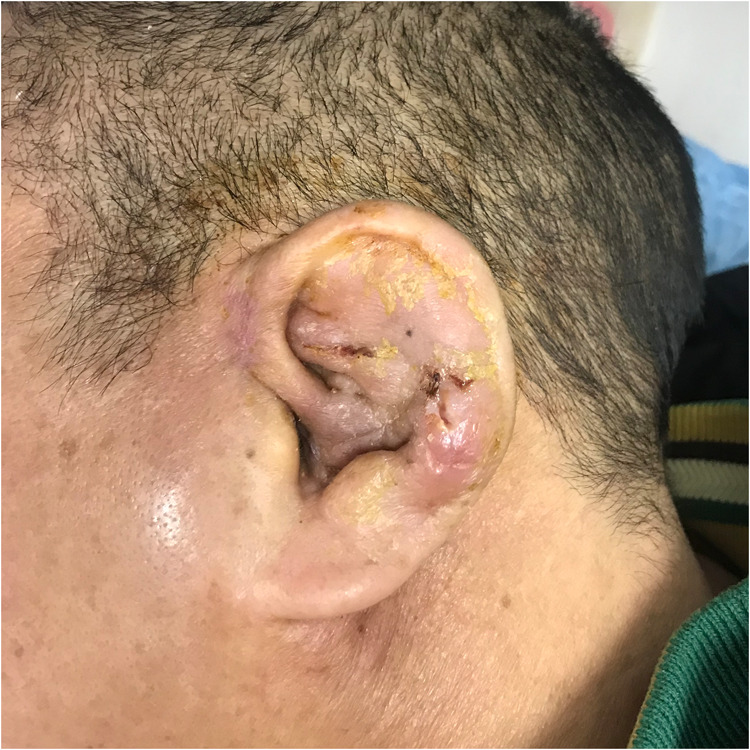
Redness and swelling of the patient's auricular skin before ultrashort wave therapy.

**Figure 2:**
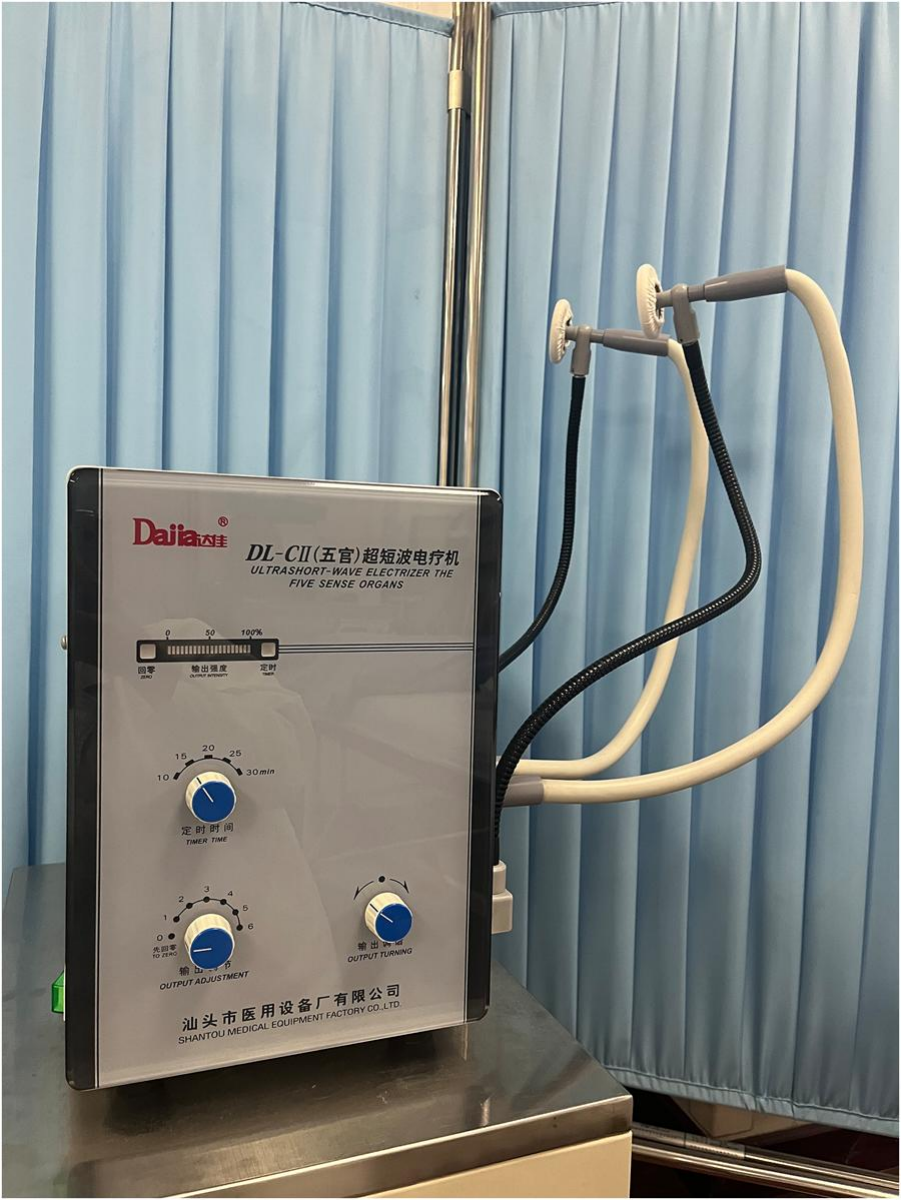
Ultrashort-wave electrizer the five sense organs.

**Figure 3:**
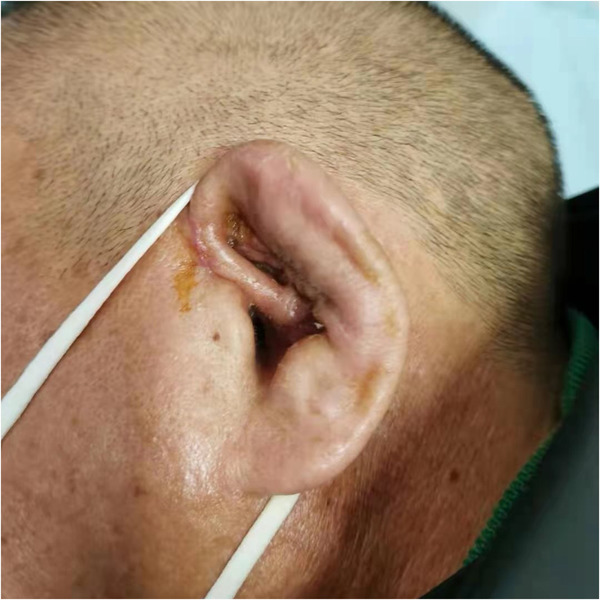
The patient's auricle has largely returned to normal appearance after 10 sessions of ultrashort wave therapy.

A 48-year-old male patient was admitted to the Department of Otolaryngology-Head and Neck Surgery at our hospital for treatment of recurrent purulent discharge from the left ear and hearing loss for over 20 years, with worsening symptoms for the past year. Admission examination revealed normal auricular morphology in both ears. The left external auditory canal showed purulent discharge with large tympanic membrane perforation. The right external auditory canal was clear with an intact tympanic membrane. Over five years ago, the patient was diagnosed with diabetes during physical examination and had consistently taken metformin and vildagliptin to control blood glucose levels. After admission, preoperative examinations were completed. After the normal control of blood glucose, the patient underwent left epitympanum and antrum opening surgery combined with tympanoplasty and canalplasty under general anesthesia. The surgical cavity was packed with gelatin sponge. The patient was discharged on the fourth postoperative day with medication. On the 10th postoperative day, the patient was readmitted due to left ear pain (VAS = 6.8). Physical examination revealed marked congestion and swelling of the left auricle with elevated skin temperature. Purulent discharge was visible within the concha, and the skin on the posterior auricle exhibited tenderness and fluctuation. The routine blood test showed a white blood cell count of 10.82 × 10^9^/L and a neutrophil percentage of 76.9%. An incision and drainage procedure was performed on the concha of the left auricle and the area behind the ear where fluctuation was most pronounced. During surgery, a small amount of white purulent discharge was observed. Culture of the specimen revealed Corynebacterium, which tested sensitive to vancomycin, meropenem, and linezolid. Intravenous administration of vancomycin for anti-infective treatment and wound drainage yielded unsatisfactory results. Subsequent testing of immune-related markers, including complement components C3 and C4, immunoglobulins, rheumatoid factor, antinuclear antibodies, and anti-double-stranded DNA antibodies, all yielded negative results. Further debridement revealed redness and swelling of the skin in the triangular fossa and antihelix of the left auricle. Upon incision, approximately 2 mL of foul-smelling white purulent discharge was drained. The surgical cavity contained inflammatory necrotic tissue and partial necrotic cartilage. The surgical debridement was thorough, with enhanced anti-inflammatory treatment and repeated dressing changes. Despite these measures, the patient's auricular skin remained red, swollen, and painful (Figure 1). The patient was referred to our hospital's Rehabilitation Department for ultrashort wave adjunctive therapy. A DL-CII type (five sense organs) ultrashort wave electrotherapy machine (Shantou Dajia Medical Equipment Co., Ltd., Shantou, Guangdong, China) was used in this study. The key parameters of the device were as follows: operating frequency of 27.12 MHz and output power of 50 W (Figure 2). During treatment, the patient sat upright with two electrodes placed opposite each other on the left and right ears, firmly adhered to the ear dressings. The treatment delivered no heat or only slight heat, with an output of 30–50 mA. Each session lasted 15 min and was performed once daily. After 10 consecutive days of treatment, the redness and swelling of the patient's auricular skin subsided, pain was alleviated (VAS = 1.3), and the appearance largely returned to normal (Figure 3).

## Discussion

Suppurative auricular perichondritis is an acute suppurative inflammation in the auricular cartilage and perichondrium. Since the auricular cartilage lacks a dedicated blood supply, once inflammation occurs, it becomes extremely difficult to control with medication. This often leads to cartilage necrosis and liquefaction, ultimately resulting in auricular deformity. Postoperative suppurative perichondritis following chronic otitis media surgery is uncommon (3, 4). It may be related to the following possible causes: 1. Surgery for otitis media typically involves concha plasty and enlargement of the external auditory canal opening. Intraoperative complications may include cartilage injury or exposure of cartilage ends. 2. Improper postoperative packing, particularly excessive tight packing at the external auditory canal opening, resulting in cartilage compression, necrosis, and infection. 3. Persistent infection within the surgical cavity.

The standard treatment for suppurative auricular perichondritis primarily involves etiological considerations and surgical intervention (5, 6). Etiological management begins with collecting evidence of the causative pathogen. Studies report that Pseudomonas aeruginosa is detected in 75%–95% of patients who develop auricular perichondritis following ear surgery, followed by Staphylococcus aureus (2, 7). The second step involves selecting appropriate antimicrobial agents based on the pathogen's susceptibility testing results. In the case presented here, Corynebacterium was cultured from the pus, but the specific bacterial species could not be identified. We selected antibiotics sensitive to the patient's antibiotic susceptibility test for treatment. Although the infection did not spread further, the reduction in local skin swelling was limited. Surgical intervention plays a crucial role in the management of suppurative auricular perichondritis (6). For patients with formed abscesses, incision and drainage should be performed promptly, with enhanced wound dressing changes. In principle, affected cartilage, perichondrium, and surrounding compromised skin should also be cleaned. However, whether to address the surgical cavity concurrently with initial drainage hinges on determining whether cartilage involvement is present. In this case, although we initially performed incision and drainage of the abscess, inflammation remained poorly controlled. This is likely because necrotic cartilage retained within the surgical cavity triggers further immune-mediated inflammatory reactions. Therefore, to prevent further progression of inflammation and destruction of more cartilage, we proceeded to thoroughly debride the surgical cavity. Postoperatively, the patient's auricle did not develop any new suppurative infections, subsequently transitioning to persistent swelling of the skin and cartilage.

Ultrashort wave therapy is a form of high-frequency electrotherapy that demonstrates effective treatment outcomes for chronic cervical and lumbar spine pain, peripheral neuritis, osteoarthritis, pulmonary inflammation, soft tissue injuries, spinal cord injuries, and cerebral ischemia-reperfusion injury (8–12). This therapy generates both thermal and non-thermal effects through high-frequency oscillating currents. The thermal effect can dilate capillaries in the treatment area, improve local blood circulation and nutrient metabolism, enhance the efficacy of intravenous medications at the site of infection, promote the clearance of inflammatory products and absorption of secretions, and accelerate wound healing (9, 10). Non-thermal effects can significantly augment the expression of iNOS and Arg-1, markers of M1 and M2 macrophages, thereby activating macrophages and promoting their phenotypic shift from M1 to M2. Concurrently, they suppress the expression levels of the inflammatory marker TNF-α, thereby lessening inflammatory responses (11). Furthermore, ultrashort wave therapy can increase Bcl-2 protein expression, thereby reducing tissue edema (12). In this case, although thorough surgical debridement and antibiotic administration effectively controlled the suppurative infection, the persistent inflammatory response and auricular skin swelling hampered wound healing within the surgical cavity. These factors represent high-risk indicators for recurrent infection. Furthermore, the pain and altered appearance of the auricle significantly impacted the patient's daily life. Ultrashort wave therapy can suppress local inflammatory responses, accelerate wound healing within the surgical cavity, reduce auricular skin edema, alleviate pain, restore normal auricular appearance, and enhance the patient's quality of life.

Cases of postoperative suppurative auricular perichondritis following chronic otitis media surgery are rare. Treatment for these patients is often limited to surgical debridement, dressing changes, and antibiotic use, resulting in prolonged and complex treatment cycles. Integrating ultrashort-wave physical therapy into rehabilitation can enhance the efficacy of surgical intervention and shorten the course of illness. For postoperative suppurative auricular perichondritis following chronic otitis media surgery, a multidisciplinary collaborative model is worthy of promotion.

## Data Availability

The original contributions presented in the study are included in the article/Supplementary Material, further inquiries can be directed to the corresponding author.
